# Effects of on-farm hatching on hatching success and first-week chick performance of local and commercial broiler genotypes

**DOI:** 10.5194/aab-69-181-2026

**Published:** 2026-03-16

**Authors:** Meryem Güler, Metin Petek

**Affiliations:** 1 Department of Animal Science, Faculty of Veterinary Medicine, University of Bursa Uludag,16059 Bursa, Türkiye

## Abstract

This study was conducted to compare conventional hatchery-hatching and on-farm-hatching systems in terms of hatching success and first-week chick performance for commercial Cobb 500, Ross 308, and local Anadolu-T genotypes. Eggs in both the conventional and on-farm-hatching groups were subjected to standard hatchery procedures until the end of 18 d of hatching. Subsequently, the eggs in the on-farm-hatching group were transported and placed in pens, while the eggs in the conventional-hatching group were transferred to the hatcher. Data on the length of the hatch window, hatching time, and other hatchery traits were collected for all groups during the hatching process. Post-hatch body weight and chick length were measured 24 h after the end of the hatch window for all groups. Chick feeding behaviour was evaluated through direct observation. There were no significant differences in terms of the hatchability of fertile eggs and total embryonic mortality between the hatching or genotype groups. However, on-farm hatching resulted in a higher chick body length at day zero ( P<0.001 ). Hatching time was significantly influenced by the hatching system ( P<0.004 ) and genotype ( P<0.001 ). The hatching system and genotype had significant effects on feed pecking behaviour at the feeder ( P<0.009 and P<0.03 , respectively). Based on these findings, it can be concluded that the on-farm-hatching system appears to be competitive with conventional-hatching systems. Despite some limitations of this study, the local broiler genotype could play a crucial role in local sustainable broiler meat production. Further study is required to evaluate growth performance and other relevant parameters under commercial conditions and in large-scale populations.

## 1 Introduction

Hatchery conditions and post-hatch environment, as well as genotypes, have a crucial impact on the post-hatch life and subsequent growth performance of chicks (de Jong et al., 2019; Hedlund et al., 2019; Erensoy et al., 2024). In commercial hatching practice, the length of the hatch window ranges between 24 and 48 h (Wang et al., 2020; Souza da Silva et al., 2021). However, chicks that hatch early often experience prolonged feed deprivation, lasting over 36 h (de Jong et al., 2017). This delay is aggravated by the prolonged transport from hatcheries to production farms, which frequently takes place without access to feed and water (Jacobs et al., 2016; de Jong et al., 2017; Erensoy et al., 2024). According to the EFSA Report on the Welfare of Domestic Birds (Nielsen et al., 2022), the maximum time to first access of feed and water must not exceed 48 h. To mitigate the adverse effects of post-hatch feed shortages on poultry health, chick development, and performance, alternative hatching systems or early-feeding practices can be implemented. However, this delay can be avoided by either facilitating on-farm hatching or providing feed and water to chicks at the hatchery (Hollemans et al., 2018; de Jong et al., 2019; Boyner et al., 2023; Madej et al., 2024).

On-farm hatching can develop chick quality (Molenaar et al., 2023), and low relative humidity does not interfere with the hatching process (Kustra et al., 2024). Chicks hatched within the farm had significant body weight development at 7 d of age and a higher survival rate during the first week of life (Rutherford et al., 2024). On-farm hatching may contribute substantially to reductions in antimicrobial use in broilers, and, therefore, it could play a crucial role in the future of more sustainable and ethical broiler meat production (Jerab et al., 2023; Boothe et al., 2024). On-farm hatching also provides extra hours for the chicks hatched late to live if compared to the traditional-hatching system (Abdullah, 2022). On-farm hatching reduces total mortality and the presence of foot pad dermatitis (de Jong et al., 2020). Currently, on-farm hatching of broiler chickens is gaining popularity in Europe, specifically in commercial systems (Vencomatic Groups, the Netherlands; One2Born, the Netherlands; Nestborn, Belgium), and has also been adopted by farms in the US, Canada, and Russia (Hein, 2024). Recently, an attempt has been made to integrate on-farm hatching with in ovo sexing for layer chickens (Yaman, 2023; Montalcini et al., 2023).

Due to breed-related factors on chick quality, it is essential to consider broiler genotype during the selection of which eggs or chicks to place for production. Egg weight, embryonic metabolic activity during incubation, and genotype are key factors influencing chick quality and first-week mortality (Vieira and Moran, 1998; Hamidu et al., 2007). Recently, a local broiler genotype named Anadolu-T was developed and launched for commercial production in Türkiye (Tagem Arge & Innovation, 2022). The first findings on the overall performance of the Anadolu-T genotype revealed it to be competitive but lower than that of commercial broilers (Erensoy and Sarıca, 2023). These broiler chicks may provide a promising basis for sustainable broiler meat production (Güler and Petek, 2024). However, there is a need for a detailed investigation and response regarding the effect of different hatching conditions and chick quality as compared to commercial genotypes. Chick responses to environmental stressors until the first access to feed and water may differ according to genotype. Therefore, this study was conducted to investigate the effects of the hatchability success rate of varying broiler genotypes in on-farm-hatching systems and their first-week chick performance under experimental conditions.

## 2 Material and methods

This study was performed in the Research and Experimental Farm of the Faculty of Veterinary Medicine in Bursa Uludag University.

### 2.1 Experimental design

The experiments were performed using a total of 599 first-grade hatching eggs (200 eggs from each genotype, except for Ross 308 with 199; see Table 1) obtained from a commercial breeder company, raising two fast-growing commercial broiler breeders (Ross 308 and Cobb 500) and a Turkish local broiler breeder (Anadolu-T) in standard conditions for broiler breeder (Ross, 2018; Cobb Vantress, 2020). The age of parent stocks used in the experiment ranged from 38 to 45 weeks between the genotypes. The averages for the individual egg weights of Anadolu-T, Cobb 500, and Ross 308 were calculated to be 58.73, 68.72, and 56.88 g, respectively.

**Table 1:** Experimental design of the study.

599 hatching eggs					
300 hatching eggs: hatchery hatching			299 hatching eggs: on-farm hatching		
Anadolu-T	Ross 308	Cobb 500	Anadolu-T	Ross 308	Cobb 500
100 hatching eggs	100 hatching eggs	100 hatching eggs	100 hatching eggs	99 hatching eggs	100 hatching eggs
81 chicks^*^	91 chicks^*^	85 chicks^*^	80 chicks^*^	87 chicks^*^	79 chicks^*^

^*^ Hatching chick number.

### 2.2 Environmental conditions and management

Fresh eggs were stored at 22–23 °C and 55 % RH for 1 d and then were weighed individually before being transferred to the setter. Eggs from all genotype groups were evenly distributed across 18 hatching trays, each with a capacity of 35 eggs, and were incubated under standard incubation conditions (Çimuka setter) until the end of 18 d of incubation. The setter was set to maintain incubation patterns at 37.2–37.7 °C and 55 % RH. Eggs were turned at an angle of 45° at a frequency of 24 times d^−1^. The room where the setter was located had an average temperature of 24 °C and 34 % RH. At embryonic day 18, half of the eggs in each genotype group were randomly assigned to conventional-hatching (HH) and on-farm-hatching (OH) treatments without candling. Thus, six treatment groups were included in the study, comprising three broiler genotypes and two hatching practices. HH eggs were moved to hatching baskets for transfer into the hatcher, whereas OH eggs remained in setter trays. The setter trays of the OH group were transferred to the farm by vehicle in under 15 min, during which time the temperature was kept stable at 24–26 °C.

The hatcher for HH eggs was set to maintain incubation patterns at 36.5–37.2 °C and 65 % RH. The experimental unit, where feed and water were available, was heated centrally by radiant tube heaters. OH eggs within egg trays were placed on a plastic slat, horizontally, side by side, 70 cm above the littered floor in each pen and 80–100 cm from the heater. At the beginning of the hatch window, egg trays in the OH group were transferred into the litter from the plastic slat. Temperature and humidity values were measured in two ways: using probes placed on the walls and using probes located ≤ 10 cm away from the egg level. During this period, the ambient relative humidity remained around 44 %–47 %, the wind speed remained below 0.15 m s^−1^, and the ambient temperature remained between 31–33 °C. At the egg level, temperature ranged from 34 to 36 °C, and relative humidity ranged from 26 % to 29 %. Continuous lighting was provided for the eggs during the hatching process in both hatching groups. The hatch window was monitored between the 470th and 510th hours of incubation in all groups using direct visual observation. The hatch window comprised the period between the first and last chick hatched in each basket (hatchery hatching) or each pen (on-farm hatching). The eggs/chicks were controlled every 2 h to count the number of hatched chicks. Eggshells were collected per basket or per pen during each control. All hatched chicks in the HH group were kept in the hatcher until the end of the hatch window. At the end of the 510th hour of incubation, unhatched eggs were collected from the basket or pen, opened, and examined macroscopically to determine fertility and hatchability rates (for both fertile and total eggs).

In the experimental growing house, a total of 30 pens were used to assess the first-week performance of the chicks, with 5 pens per group and a maximum of 15 chicks per pen. The experimental pens were 1 m × 1 m in size and equipped with nipple drinkers and chick feeders. They were subjected to the same care and management programme as specified in Turkish legislation for the protection of meat-type poultry (Official Gazette, 2018). The birds received 24 h of artificial light for the first 7 d. No feed and water were provided to the hatchery-hatched chicks until the first access to feed and water, which was 24 h after the hatch window. Subsequently, feed and water were provided ad libitum. All birds in every group received the same feed ad libitum, consisting of maize- and soybean-meal-based commercial diets formulated to meet their nutritional requirements (starter: crude protein = 22 %, metabolisable energy = 2900 kcal kg^−1^). The feed was provided in crumble form, with a feeder space of approximately 2.5 cm per chick, initially.

### 2.3 Data

#### 2.3.1 Hatchability

The hatchability of fertile or total eggs was calculated as the number of chicks hatched per fertile or total egg set, and the fertility results were reported as “apparent fertility”. Chicks hatched between the 470th and 484th hours of incubation were classified as early, those hatched between the 485th and 501st hours were classified as mid-term, and those hatched between the 502nd and 510th hours were classified as late. Total embryonic mortality was calculated as a percentage of the total number of fertile eggs. In this study, the period between the end of the hatch window and the first access to feed and water (24 h later) was defined as day zero as this interval is typically spent in the hatchery or in transport under commercial conditions. The first feed and water were provided to the HH chicks 24 h after placement in the research facility, while OH chicks were provided with feed and water as soon as they hatched. Feed was supplied on chick papers and in chick feeders during the first 2 d in all groups. All chicks in all groups were individually weighed and their body lengths were measured 24 h after hatching was completed. Chick body length was measured by stretching the chick along a ruler and taking the range from the tip of the beak to the tip of the right middle toe, excluding the nail (Petek et al., 2008, 2010; Souza da Silva et al., 2021).

#### 2.3.2 Chick feeding behaviour

After providing immediate access to feed and water for all chicks in both groups, the feeding behaviour of chicks was monitored through direct observation in every group. Observations were conducted at 2 h intervals throughout the first day. During each observation period, an experienced researcher recorded the number of birds in each group exhibiting specific behaviour, including pecking at feed in the feeder, pecking at the litter or other objects on it, and remaining in close proximity to the feeder. Collected data were then used to calculate the frequency of birds with a particular behaviour indicator as a proportion of the total number of birds in the respective group.

### 2.4 Statistical analysis

The collected data for hatchability, day-old chick weight, day-old body length, and mortality were statistically analysed using the SPSS 28.0 statistical package (IBM Corp., 2021) by means of a generalised linear model with the hatching system (conventional hatchery hatching and on-farm hatching) and broiler genotype (Anadolu-T, Cobb 500 and Ross 308) as the main effects, along with all of the interactions between the two main factors according to a normality distribution (Snedecor and Cochran, 1991; Salinas Ruíz et al., 2023). Duncan's multiple-range test was used for post hoc comparisons between the groups when necessary. Based on the sample size and data distribution, no data transformation was required for proportional data to stabilise variance or normalise the data (Lin and Xu, 2020). Day-zero chick mortality included all birds that died from hatching until the end of the 24 h feed deprivation period of HH chicks after placement on the farm. It was calculated as a percentage of the post-hatch live-chick number ((dead and second-quality chicks) / post-hatch live chick number) and was not included in first-week chick mortality. First-week chick mortality included all birds that died from 1 d old to the end of the first week of age. Exact p values are reported in the tables and were compared with the predefined significance level ( α= 0.05).

## 3 Results

The effects of different hatching systems on the hatching performance of different broiler genotypes are presented in Table 2. There were significant differences in the hatchability of total eggs ( P<0.05 ), apparent fertility ( P<0.05 ), and average hatching time ( P<0.001 ) between the hatching groups. The hatchability of total eggs ( P<0.05 ), total embryonic mortality ( P<0.05 ), and length of hatching time ( P<0.001 ) were found to be significantly different among genotypes. The length of the hatch window in all experimental groups was to be varied from 32 to 36 h. The average hatching time was the lowest in the HH × Anadolu-T group and was the highest in the OH × Cobb 500 group. There were no significant differences in the hatching system × genotype interaction for any hatching performance parameters.

**Figure 1:**
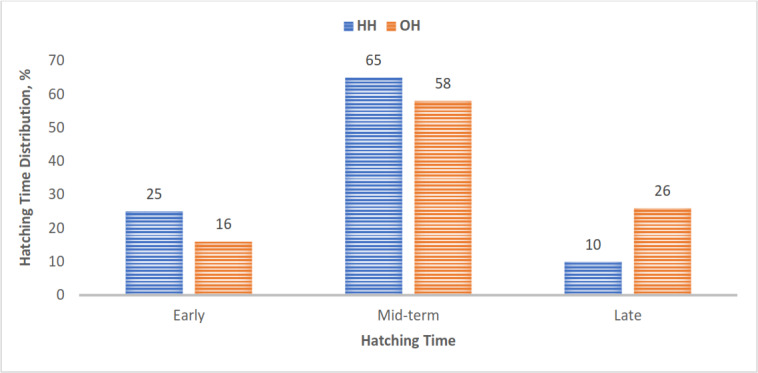
Hatching-time distribution of chicks in the hatching groups. Bars represent the number of chicks hatched early, mid-term, and late in each hatching group (HH represents hatchery hatching, and OH represents on-farm hatching).

In the study, 65 % and 58 % of chicks in the HH and OH hatching groups, respectively, hatched during a mid-term incubation period of 484 and 501 h. The HH group had a greater percentage of early-hatched chicks and a lower percentage of late-hatched chicks than the OH group (Fig. 1).

**Figure 2:**
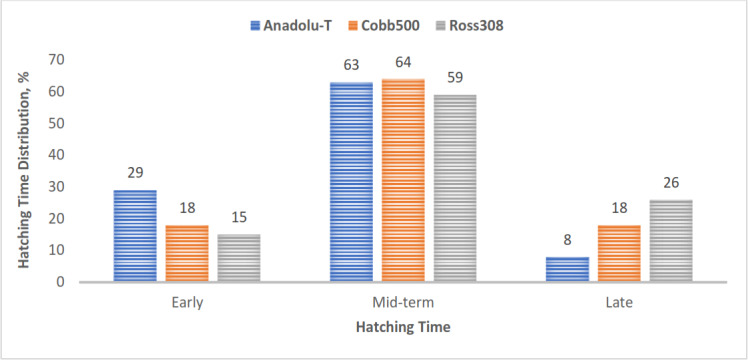
Hatching-time distribution in the genotype groups. Bars represent the number of chicks hatched early, mid-term, and late per genotype.

In terms of genotype, 59 %–64 % of chicks from all genotypes hatched during the mid-time interval of 485 to 501 h of incubation. The Anadolu-T group had a higher percentage of early-hatched chicks, whereas Ross 308 had a higher percentage of late-hatched chicks (Fig. 2).

**Figure 3:**
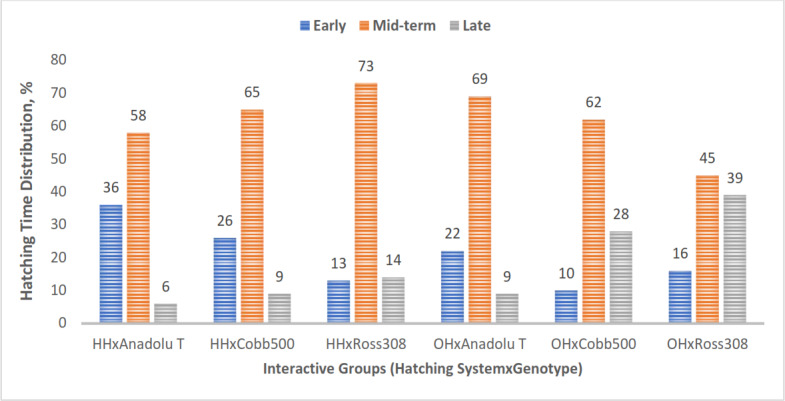
Hatching-time distribution of chicks in the experimental groups. Bars represent the number of chicks hatched early, mid-term, and late in each experimental group (HH represents hatchery hatched, and OH represents on-farm hatched).

In all experimental groups, the majority of chicks hatched between 485 and 501 h of incubation, corresponding to the mid-term period (Fig. 3). The lowest number of chicks hatched during this interval was observed in the OH × Ross 308 group (45 %), while the highest number of chicks was recorded in the HH × Ross 308 group (73 %).

Day-old chick body weight, day-old chick length, and mortality rate among the groups are presented in Table 3. There were significant differences in the day-old chick body weight and chick body length between hatching ( P<0.001 ) or genotype ( P<0.001 ) groups. Chicks from Cobb 500 breeder hens had significantly greater body weight and longer body length at day-old age than those of Anadolu-T and Ross 308. The average body weights of HH and OH chicks were found to be 42.94 and 44.02 g, while body lengths were 16.89 and 17.87 cm, respectively. The hatching system and genotype were significantly influenced by day-zero and first-week chick mortality ( P<0.05 ).

**Table 2:** Effects of hatching systems on the hatching performance of eggs from different broiler genotypes.

Groups	Hatchability of total	Apparent fertility	Hatchability of fertile	Total embryonic	Hatch window	Hatching time^*^
	eggs %	%	eggs %	mortality %	h	h
Hatching system						
HH	85.66	91.66	93.41	6.58	36.00	494.06 ± 0.58
OH	82.29	88.36	92.80	7.19	33.30	496.86 ± 0.60
Genotype						
Anadolu T	80.50^b^	89.00^b^	90.45^c^	9.55^a^	34.00	492.08 ± 0.73^b^
Cobb 500	82.00^b^	88.00^b^	93.15^b^	6.84^b^	35.00	496.50 ± 0.73^a^
Ross 308	89.44^a^	93.05^a^	95.72^a^	4.27^c^	35.00	497.80 ± 0.70^a^
Hatching system × genotype						
HH × Anadolu T	81.00	89.00	91.01	8.98	36.00	490.21 ± 1.03
HH × Cobb 500	85.00	90.00	94.44	5.55	36.00	494.17 ± 1.03
HH × Ross 308	91.00	96.00	94.79	5.20	36.00	497.80 ± 1.00
OH × Anadolu T	80.00	89.00	89.88	10.11	32.00	493.95 ± 1.04
OH × Cobb 500	79.00	86.00	91.86	8.14	34.00	498.82 ± 1.05
OH × Ross 308	87.87	90.09	96.66	3.33	34.00	497.80 ± 1.00
P value						
Hatching system	0.035	0.002	0.496	0.494	0.001	0.004
Genotype	0.001	0.001	0.001	0.001	0.241	0.001
Hatching system × genotype	0.060	0.099	0.121	0.990	0.194	0.152

^*^ Average hatching time (mean ± SE). ^a–c^ Significant differences among the groups in the same row.

**Table 3:** Effects of hatching systems on day-old chick body weight (mean ± SD), day-old chick body length (mean ± SD), and day-zero and first-week chick mortality (%) of different broiler genotypes.

Groups	Day-old chick body	Day-old chick body	Day-zero chick	First week chick
	weight (g)	length (cm)	mortality^*^ (%)	mortality^**^ (%)
Hatching system				
HH	42.94 ± 0.252	16.89 ± 0.049	0.75	0.82
OH	44.02 ± 0.264	17.87 ± 0.045	5.16	5.71
Genotype				
Anadolu T	41.19 ± 0.300^b^	17.29 ± 0.052^b^	0.62^b^	4.39
Cobb 500	48.63 ± 0.295^a^	17.85 ± 0.054^a^	7.12^a^	3.68
Ross 308	41.13 ± 0.270^b^	17.05 ± 0.050^b^	1.12^b^	1.74
Hatching system × genotype				
HH × Anadolu T	40.35 ± 0.376	16.61 ± 0.067	0	2.46
HH × Cobb 500	48.01 ± 0.379	17.60 ± 0.073	1.17	0
HH × Ross 308	40.67 ± 0.285	16.54 ± 0.073	1.09	0
OH × Anadolu T	42.02 ± 0.378	18.02 ± 0.075	1.25	6.32
OH × Cobb 500	49.34 ± 0.496	18.04 ± 0.059	13.9	7.35
OH × Ross 308	41.12 ± 0.343	17.59 ± 0.057	1.14	3.48
P value				
Genotype	0.001	0.001	0.001	0.100
Hatching	0.001	0.001	0.011	0.001
Genotype × hatching	0.590	0.001	0.099	0.152

^*^ Day-zero chick mortality included all birds that died from hatching until the end of the 24 h feed deprivation period of hatchery-hatched chicks after placement on the farm. It was calculated as a percentage of post-hatch live-chick number (dead and second quality chicks/post-hatch live chick number) and did not include first-week chick mortality. ^**^ First-week chick mortality included all birds that died from day-old age until the end of the first week of age. It was calculated as a percentage of the day-old chick number (end-of first-week chick number / day-old chick number). ^a,b^ Means with different letters in the same colums are statistically important.

Different feeding behaviours among the groups are shown in Table 4. There were significant differences in the distribution of chicks pecking feed from the feeder and pecking ground or litter among the genotype groups. No significant differences were observed in the number of chicks close to the feeder between the hatching systems or genotype groups. There were significant effects of hatching system and genotype on the total feed-related behaviour of chicks ( P<0.05 ).

**Table 4:** Distribution of chicks showing different kinds of feeding behaviour as a percentage of total birds in the groups (mean ± SD).

Groups	Birds pecking feed	Chicks close to	Chicks pecking	Total feed-related
	on the feeder	the feeder	ground or litter	behaviour
Hatching system				
Hatchery hatch (HH)	37.10 ± 1.6	20.05 ± 1.0	18.61 ± 1.4	75.60 ± 1.6
On-farm hatch (OH)	18.03 ± 1.7	22.47 ± 1.6	13.32 ± 1.2	50.49 ± 1.9
Genotype				
Anadolu-T	38.31 ± 1.0^a^	23.56 ± 1.4	9.34 ± 2.0^b^	65.97 ± 2.6^b^
Cobb 500	15.10 ± 1.1^b^	15.11 ± 3.0	14.28 ± 1.1^ab^	44.49 ± 2.4^c^
Ross 308	29.29 ± 1.5^ab^	25.11 ± 1.4	24.28 ± 1.6^a^	78.68 ± 1.9^a^
Hatching system × genotype				
HH × Anadolu T	54.11 ± 3.0	14.63 ± 1.2	4.92 ± 1.0	73.99 ± 2.6
HH × Cobb 500	24.49 ± 1.7	24.71 ± 3.0	19.99 ± 1.3	69.19 ± 3.2
HH × Ross 308	32.70 ± 7.0	20.81 ± 1.1	30.91 ± 4.0	84.42 ± 3.5
OH × Anadolu T	22.50 ± 1.3	32.50 ± 1.2	13.75 ± 1.4	58.75 ± 3.3
OH × Cobb 500	5.71 ± 1.0	5.52 ± 2.0	8.56 ± 2.0	19.79 ± 3.3
OH × Ross 308	25.88 ± 2.4	29.41 ± 1.5	17.64 ± 1.3	72.93 ± 3.4
P value				
Hatching system	0.009	0.545	0.184	0.001
Genotype	0.030	0.106	0.014	0.050
Hatching system × genotype	0.335	0.062	0.052	0.090

^a–c^ Significant differences among the groups in the same row.

## 4 Discussion

In this study, we investigated the effects of the on-farm-hatching system on the hatching success and early-period chick performance of three different broiler genotypes. There were no significant differences for the hatchability of fertile eggs (hatching rate) and total embryonic mortality, which are accepted as the main indicators of the effectiveness of a hatching system in practical conditions, between the conventional hatchery-hatched (HH) and on-farm-hatched (OH) groups. This was similar to some of the previous findings on the effects of early feeding or on-farm-hatching systems on hatching performance. On the other hand, the hatchability of total eggs and the apparent fertility rate in the OH group were found to be significantly lower than in the HH group, while the hatching time of on-farm-hatched chicks was found to be significantly longer. The lack of a difference in terms of the hatchability of fertile eggs suggests that OH can be used instead of HH in the production. However, the low hatchability of total eggs and the low apparent fertility rate suggest that these traits may have been influenced by unknown or unpredictable factors before hatching and during the first 18 d of incubation. In this study, the hatch window was found to be shorter in the OH group compared to in the HH group. The Ross 308 genotype group had a significantly greater hatchability of total and fertile eggs, a greater apparent fertility rate, and the lowest embryo mortality than those of the other two genotype groups. These rates were found to be better in the HH × Ross 308 group compared to in the OH × Ross 308 group. Contrarily to our findings, hatchability was reported to be, on average, 2.5 % higher with the X-Treck on-farm-hatching system (Hein, 2024). In that study, similarly to our results, Ross flocks exhibited slightly higher hatchability than Cobb flocks with the X-Treck system. In a semi-experimental study using the One2Born on-farm-hatching system, Collin et al. (2024) found that the hatching rate of on-farm-hatching groups (90.4 %) was slightly lower than that of conventional-hatching groups (94.0 %). Kustra et al. (2024) reported that the hatchability of the HH group was 96.4 % compared with 93.9 % and 95.8 % for OL (on-farm hatching on litter floor) and OT (on-farm hatching on plastic floor), respectively. Local Anadolu-T had the greatest embryonic mortality rate among the genotype groups.

Maintaining a stable on-farm-hatching environment, especially in-pen or floor temperature and relative humidity, is crucial for achieving results comparable to conventional hatchery systems. In practice, the average temperature in on-farm-hatching conditions is usually 3–6 °C lower than in conventional hatchery hatching (Boyner et al., 2021; Witjes et al., 2022; Kustra et al., 2024). The lower temperature during on-farm hatching might be beneficial for chick homeostasis (de Jong et al., 2020). The average relative humidity around the eggs during on-farm hatching is generally lower than that under hatchery conditions. Low relative humidity during hatching can reduce hatchability; however, it may enhance albumen utilisation (Boleli et al., 2016; Kustra et al., 2024) and promote drying of eggshell membranes (Van der Pol et al., 2013).

In this study, the hatch window of OH chicks was determined to be significantly (2.67 h) shorter than that of HH chicks. A short hatch window is essential in the commercial poultry industry to achieve high chick uniformity and improved chick quality (Zhong et al., 2018). Generally, a hatch window of 24 h is considered to be optimal in poultry production; however, a duration of up to 36 h is frequently observed. Embryo temperature during incubation and egg size are the most important factors influencing the length of the hatch window (Tona et al., 2022). Eggs stored for 12 d exhibited significantly shorter hatch windows than those stored for 0 to 9 d (Abioja et al., 2022). A hatch window ranging from 24 to 48 h is known to affect post-hatch performance, corresponding to 5 %–10 % of total embryonic development (Tong et al., 2013; Wang et al., 2020). Monitoring the hatch window is highly beneficial for determining the optimal time for chick collection from the hatcher. Chicks that hatched earlier may spend a longer time without access to feed and water; in combination with post-hatch handling and transportation, all of these factors may contribute to transport-related mortality (Xin and Lee, 1997).

In this study, the majority of chicks in both hatching systems hatched around day 20 of incubation, corresponding to 484–501 h. There was a significant difference in total hatching time between the hatching system and genotype groups. Chicks hatched conventionally and those from the Anadolu-T genotype had significantly shorter hatching times. The length of hatching time affects chick quality and post-hatch chick performance (Dişa et al., 2022). Such et al. (2023) reported that the average hatching times were 489.4 and 493.9 h for the early- and late-hatched groups, respectively. In the present study, the first chick across all genotypes in the HH system hatched at 474 h of incubation. Regarding genotype, the first chick hatched at 478 h in the Anadolu-T × OH group, whereas the first chicks of Ross 308 and Cobb 500 hatched at 476 h in the OH system. The peak hatching time in all groups was observed between 485 and 501 h of incubation (Fig. 2). The proportion of chicks hatching at peak time was relatively lower in the Anadolu-T genotype and higher in the HH group. The reason for this is likely to be that the in-pen air temperature during the on-farm-hatching process was slightly lower and likely more variable than required and not uniformly distributed across all egg surfaces. We were not able to measure eggshell temperature during the on-farm-hatching process to clarify this. However, we tried to set up a stable air temperature and RH over the eggs for all experimental pens during the hatching process and subsequent period. In-pen or in-house temperature remains almost constant during the hatching and subsequent growth periods. It is worth noting that other factors in the on-farm-hatching process may also impact hatching success, such as the height of the eggs above the ground, ground insulation, and other pre-incubation factors (Abioja et al., 2022). Incubation parameters have varying effects on post-incubation performance parameters. Hatching time alters the bacterial composition of the ceca in the early life of chicks due to differences in bacterial colonisation in the hatcher and differences in access to feed and water between early- and late-hatched chickens (Such et al., 2023). Regardless of egg weight, there was a negative correlation between hatching time and chick body weight from day 4 onward and throughout the experiment (Løtvedt and Jensen, 2014). Late-hatching chicks were significantly heavier than early and mid-term hatchlings, but, by 3 d of age, early hatchlings were heavier than mid-term and late hatchlings (Boyner et al. 2021). Groves and Muir (2017) reported that Cobb chicks hatching at ≤ 498 h of incubation grew faster during the first 7 d than those that hatched later. There were no significant differences in embryonic mortality between the hatching groups. These findings may suggest that variations in pre-hatching environmental conditions do not appear to affect the survival of broiler embryos despite differences in air speed, air temperature, relative humidity, and CO_2_ concentrations surrounding the eggs in both hatching systems. Total embryonic mortality was found to be significantly lowest in Ross 308 eggs and significantly greatest in Anadolu-T eggs. Similarly to our results, Fathi et al. (2022) reported significant differences in all mortality types among the native genotypes.

The Tona score or Pasgar score, chick weight at 1 d of age, yolk-free body mass, and chick length are major quantitative methods for assessing chick quality (İpek and Sözcü, 2013). However, the Pasgar or Tona scoring systems are time-consuming as a minimum of 30 and 44 chicks, respectively, must be evaluated to obtain a representative value (Boerjan, 2006; Molenaar et al., 2023). Van de Ven et al. (2012) found no correlation between the score and either post-hatch growth or mortality in first-grade chicks. In that study, navel condition was the only Pasgar score criterion that affected the weight of 7 d old chicks. Similarly, Willemsen et al. (2008) reported no correlation between the Tona score and post-hatch performance. Under field conditions, first-week mortality is commonly used to assess chick quality and production performance; however, it provides delayed information. Therefore, in terms of rapid and practical evaluation, day-old chick weight and chick length are considered to be the most reliable methods for assessing post-hatch chick quality. The live body weight of day-old chicks is crucial because it determines the growth rate of birds for the entire fattening period and is one of the key characteristics of day-old chick quality (Molenaar et al., 2023; Such et al., 2023). In this study, day-old chick body weight and body length were significantly higher in the OH group than in the HH group. Besides the hatching factor, even though the eggs are distributed equally among the hatching groups, differences in the weight of the eggs from which chicks hatch may also contribute to this. In previous reports by de Jong et al. (2019, 2020), it was found that the day-old body weights of OH chicks from each of the three genotypes were greater than those of conventional HH chicks. This difference was likely due to the earlier access to feed and water for OH chicks, resulting in the earlier development of their gastrointestinal system (Souza da Silva et al., 2021). The contribution of feed present in the gizzard and intestines to chick body weight should be taken into account, along with the effects of earlier access to feed and water in on-farm-hatched chicks. Similarly to these findings, Guilloteau et al. (2024) reported that the body weights of OH chicks were significantly greater than those of conventional HH chicks and that the OH system was at least equivalent to the conventional-hatching system. In another study, Rutherford et al. (2024) also demonstrated that OH chicks had higher body weights compared to those hatched under hatchery conditions. Consistently with our results, Molenaar et al. (2023) observed a beneficial effect of on-farm-hatching systems on post-hatch quality and the body weight of chicks derived from young breeders' eggs. In that study, it was found that the body weight of OH chicks was significantly greater than that of HH chicks, while there were no significant differences in chick body length between chicks from both hatching groups. Similarly to these findings, Jessen et al. (2021a) also showed that OH increased the body weight of chicks in the first 24 h of life. In a current study, day-old OH chickens were significantly longer than hatchery-hatched chicks, suggesting better day-old chick quality and possibly also better post-hatch performance. In terms of chick quality, Hein (2024) reported that the OH chicks had a worse naval and hock score at day 0 compared to the score of the chicks that were hatched in the hatchery. Reduced chick quality, higher second-grade chicks, and higher first-week mortality may be potential risk factors associated with on-farm-hatching applications (Jessen et al., 2021b; Riber and de Jong, 2023). In support of this finding, lower first-week and total mortality rates have been observed in both fast-growing and slower-growing broiler chickens hatched on-farm compared to in hatchery-hatched chickens (Hollemans et al., 2018; de Jong et al., 2020; Jessen et al., 2021b). In general, Cobb 500 chickens have significantly greater hatch body weight than Anadolu-T and Ross 308 chicks, primarily due to their larger hatch eggs. Similarly, day-old Cobb 500 chicks were significantly longer than Anadolu-T and Ross 308 chicks. Greater body length indicates more efficient transformation of nutritive materials in the chick's body and may positively correlate with post-hatch performance (Mukhtar et al., 2013; Mesquita et al., 2021). In addition to egg weight, other factors may affect day-old chick body length. For example, Abioja et al. (2023) reported that chicks from eggs stored for 3 d (17.3 cm) were longer than those from eggs stored for 5 (17.1 cm) or 7 d (17.1 cm), while chicks from eggs stored for 16 d were shorter (Abioja et al., 2023). In our study, day-zero and first-week chick mortality rates were found to be significantly different between the hatching and genotype groups. All on-farm-hatched chicks from all genotypes had higher day-old and first-week chick mortality compared to their counterparts of hatchery-hatched chicks. The Anadolu-T group exhibited a higher first-week mortality rate, whereas Cobb 500 showed significantly greater day-zero chick mortality. High chick mortality is a serious issue that can markedly affect production and profitability and may result from pre-incubation factors, conditions throughout the incubation period, suboptimal environmental conditions, inadequate nutrition, insufficient hygiene, or improper post-hatch care. In our study, it can be said that prolonged hatching time and slightly suboptimal brooder temperature in the pen during and after the hatching process were likely to be the main contributors, possibly due to stress associated with staggered hatching. The HH Anadolu-T group had no day-zero chick mortality, while the HH Cobb 500 and the HH Ross 308 groups had no mortalities during the period of the first week of life. In a study, it was shown that a decrease in first-week mortality of 0.24 % was observed for X-Treck OH chicks compared to HH chicks (Hein, 2024). de Jong et al. (2019) showed that body weight and first-week chick mortality were similar for both on-farm-hatching and conventional-hatching groups. In our study, day-zero chick mortality including all birds that died from hatching until the end of the 24 h feed deprivation period or until first access to feed and water was described as dead on arrival (DOA) and served as a relevant indicator for assessing chick welfare during the pre-placement holding period. Elibol et al. (2023) reported that the DOA ranged from 0.005 % to 0.107 % according to pre-placement holding time.

Providing feed and water immediately after hatching is essential to ensure high-quality day-old chicks and enhanced performance (Yeboah et al., 2019). Whether animals receive sufficient feed and water after hatching can be assessed by observing their feeding activity and checking crop fullness. There were significant differences in feeding behaviour between HH and OH chicks. A greater number of HH chicks were significantly more active in feed consumption, with more than 75 % of HH chicks exhibiting total feed-related behaviour during the observation period. This finding aligns with the findings of Giersberg et al. (2023), who reported that hatchery-hatched chicks showed higher activity than on-farm-hatched chicks. The differences in feeding behaviour between the hatching groups may be attributed to the long-term lack of feed and water for hatchery-hatched chicks due to the extended hatch window (36 h) and the post-hatch waiting period (24 h) before access to feed and water. OH chicks exhibited less eating-related activity because they had already had access to feed for 24 h. Contrarily to our findings, Jessen et al. (2021a) reported that feeding behaviour was more frequently observed in OH chicks than in HH chicks at 11 h and 35 h of the post-hatch period. In fact, at the beginning of life, chicks remain inactive for a considerable time before engaging in feeding-related activities. Boyner et al. (2021) reported that only 5 % of chicks were observed eating or standing near the feeder in the first 25.4 h of life, and 50 % of birds had a full crop by an average age of 30.6 h. Under normal hatchery conditions, the shorter the hatch window period, the more active the chicks are (Boyner et al., 2021). Regardless of genotype, the number of birds pecking feed and exhibiting total feed-related behaviour was greater in HH groups across all genotypes. The main reason for the differences in feeding behaviour between the two hatching systems was the very low activity in the OH × Cobb 500 group. Overall, more than 80 % of chicks displayed non-feed-related behaviour during the observation in this group, whereas total feed-related behaviour was observed in more than 58 % of chicks in all other groups (Table 3). This group had the highest day-zero mortality rate (13.9) and the lowest feeding activity (19.79 %), which may be due to the prolonged hatching time and the associated stress, leading to reduced chick quality. Interestingly, the chick quality was the highest in the OH × Cobb 500 group. The fact that this group also had the lowest hatchability of total eggs (79 %) and apparent fertility rate (86 %) suggests that some unpredictable factors before hatching and during the first 18 d of incubation may have had an effect on post-hatch performance.

We were unable to differentiate chicks by sex at hatch; therefore, our results reflect the first-week performance of mixed-sex chicks. We tried to provide optimal conditions for eggs and chicks throughout the experiment, from the hatchery to the farm. However, many environmental factors – particularly under open-air conditions on the farm – contribute to variations in eggshell temperature, which in turn can affect hatch window and hatching-time differences between groups. Both floor and eggshell temperatures are critical factors for ensuring a good start for day-old chicks. However, we were unable to measure either eggshell or floor temperatures during the on-farm-hatching process.

## 5 Conclusions

In this study, we investigated on-farm hatching without using any specialised equipment. Eggs were initially placed on slats above the ground and subsequently transferred to the floor. Despite certain limitations, such as variation in breeder age, the overall results of this study provide valuable insights. The on-farm-hatching system appears to be competitive with conventional-hatching systems, and on-farm hatching may provide a beneficial starting point in broiler production. The hatching and first-week performance of local Anadolu-T chicks appear to be promising, particularly for local broiler meat production; however, additional studies are needed to evaluate growth performance and other relevant parameters under commercial conditions and in larger populations.

## Data Availability

The datasets generated during this study are not currently available because the PhD research is still in progress but are available from the corresponding author upon reasonable request.
